# Comparison of Sanger sequencing for hepatitis C virus genotyping with a commercial line probe assay in a tertiary hospital

**DOI:** 10.1186/s12879-019-4386-4

**Published:** 2019-08-22

**Authors:** Sylvie Goletti, Siméon Zuyten, Léonie Goeminne, Chris Verhofstede, Hector Rodriguez-Villalobos, Monique Bodeus, Peter Stärkel, Yves Horsmans, Benoît Kabamba-Mukadi

**Affiliations:** 1 Microbiology Department, Cliniques universitaires St Luc, Université catholique de Louvain, Brussels, Belgium; 2 HepatoGastroenterology Department, Cliniques universitaires St Luc, Université catholique de Louvain, Brussels, Belgium; 3 Pôle de Microbiologie médicale, Institut de Recherche Expérimentale et Clinique, Université catholique de Louvain , Brussels, Belgium; 4 AIDS Reference Laboratory, Clinical Chemistry, Microbiology and Immunology Department, Ghent University, Ghent, Belgium

**Keywords:** Hepatitis C virus, Genotyping, Sequencing

## Abstract

**Background:**

The technique most frequently used to genotype HCV is quantitative RT-PCR. This technique is unable to provide an accurate genotype/subtype for many samples; we decided to develop an in-house method with the goal of accurately identifying the genotype of all samples. As a Belgium National Centre of reference for hepatitis, we developed in-house sequencing not only for 5’UTR and core regions starting from VERSANT LiPA amplicons but also for NS5B regions. The sequencing of VERSANT LiPA amplicons might be useful for many laboratories worldwide using the VERSANT LiPA assay to overcome undetermined results.

**Methods:**

100 samples from Hepatitis C virus infected patients analysed by the VERSANT HCV Genotype 2.0 LiPA Assay covering frequent HCV types and subtypes were included in this study. NS5B, 5’UTR and Core home-made sequencing were then performed on these samples. The sequences obtained were compared with the HCV genomic BLAST bank.

**Results:**

All the samples were characterised by the VERSANT LiPA assay (8 G1a, 17 G1b, 6 G2, 11 G3, 13 G4, and 10 G6). It was not possible to discriminate between G6 and G1 by the VERSANT LiPA assay for 8 samples and 27 had an undetermined genotype. Forty-one samples were sequenced for the three regions: NS5B, 5’UTR and Core. Twenty-three samples were sequenced for two regions: 5′ UTR and Core and 36 samples were sequenced only for NS5B. Of the 100 samples included, 64 samples were analysed for 5’UTR and Core sequencing and 79 samples were analysed for NS5B sequencing. The global agreement between VERSANT LiPA assay and sequencing was greater than 95%.

**Conclusions:**

In this study, we describe a new, original method to confirm HCV genotypes of samples not discriminated by a commercial assay, using amplicons already obtained by the screening method, here the VERSANT LiPA assay. This method thus saves one step if a confirmation assay is needed and might be of usefulness for many laboratories worldwide performing VERSANT LiPA assay in particular.

## Background

Due to its high rate of mutation, hepatitis C virus (HCV) forms viral quasispecies, classified based on the highly variable regions in the envelope protein and nonstructural 5B protein (NS5B). As a result of this large genetic variability, seven genotypes and more than 67 subtypes have been identified [1]. The global repartition of genotypes varies across countries but genotype 1 is the most common with more than 45% worldwide, followed by genotype 3 [2]. In Belgium, a recent publication showed the same distribution of HCV genotypes with 53.6% of genotype 1 (31.6% subtype 1b and 19.7% subtype 1a) followed by genotype 3 with 22.0% [3].

HCV genotyping is part of the evaluation of newly diagnosed patients and has always been important in guiding treatment [4]. In the last EASL recommendations for the treatment of hepatitis C [5], identification of HCV genotype remains an essential consideration. Since the development of pan-genotype direct acting agents (DAA), correct identification of the genotype is important to guide the choice of the DAA combination and the duration of the treatment. Indeed, several studies have shown that a misclassification of the HCV genotype can lead to therapeutic failure. Starace et al. showed that 14.9% of DAA failure are related to a genotyping error [6] and Di Maio et al. report that 6/197 (3%) of DAA-failing patients and in particular 4/7 non responders to a DAA INF-free regimen were impacted on a wrong genotype assignment [7].

In addition to its impact on the choice of treatment, genotyping is also important for epidemiological information and the development of cost-effectiveness national treatment strategies [8].

Considering that genotype is a key element in the management of patients infected with hepatitis c virus, the determination of the genotype is recommended but in recent guidelines of the European Association of the Study of the Liver (EASL) published in 2018 [5], no recommendation was made on the assay to use. It is only mentioned that “the most widely used method is based on reverse hybridization with the line probe assay” and that “a kit based on deep sequencing will soon be available” [5]. Guidelines also mentioned that genotype assays should target the 5′ untranslated region (5’UTR) and another region for identification of the genotype 1 subtype (1a or 1b), generally the core or the NS5B region [5]. In the EASL 2018 guidelines [5], it is recommended that genotypes 1(G1), 2 (G2), 3 (G3), 4 (G4), 5 (G5) and 6 (G6) and the subtypes a and b of genotype 1 (G1a and G1b) should be distinguished. Therefore, we focused this work only on subtyping G1, no analysis was done for the subtype of the other genotypes.

As mentioned above, the technique most frequently used to genotype HCV is reverse transcriptase PCR (RT-PCR), with VERSANT HCV Genotype 2.0 Line Probe Assay (VERSANT LiPA; Healthcare SIEMENS, Munich, Germany), the other most widely used commercial genotyping assays is the Abbott RealTime HCV Genotype II (Abbott GTII; Abbott, Illinois, USA) [8].

The two commercial assays detect genotypes 1 to 6 and subtypes 1a and 1b. The Abbott GTII assay targets the 5’UTR region for classification of HCV genotypes and the NS5B region for detection of the genotype 1 subtypes. The VERSANT LiPA assay targets the 5’UTR region for the genotype and the core region for subtype 1a and 1b.

These commercial assays have shown some limitations that must be kept in mind. The first limitation is the percentage of non-subtypable G1. Some publications reported that Genotype 1 subtypes were not identified by the Abbott GTII assay in 3.7 to 15.9% of cases (with a mean of 8.5%) [9–11] and in 2.2 to 7.4% of cases with the VERSANT LiPA assay [10]. Other limitations are that the commercial assays are not always able to differentiate between G6 and G1b [10], coinfection [12] or recombination of HCV genotypes [13]. In addition, an undetermined result is obtained for some samples. In our experience, over a 2-year period (2016–2017) and 678 genotype analyses performed with the VERSANT LiPA assay in the laboratory at the Cliniques Universitaires Saint-Luc, a tertiary hospital, out of a total of 276 G1b samples, 6% (17/276) could not be distinguished from genotype 6. In addition 3.5% (24/678) of analyzed samples gave an undetermined genotype during the same period. Noppornpanth et al. reported similar results in their study [14].

Because the VERSANT LiPA was unable to provide an accurate genotype/subtype for a significant number of samples, we decided to develop an in-house method with the goal of accurately identifying the genotype for all tested samples. As a National Centre of reference for hepatitis in Belgium, we decided to develop a homebrewed sequencing assay for 5’UTR and core regions starting from VERSANT LiPA amplicons. To our knowledge, this is the first description of the sequencing of VERSANT LiPA amplicons that might be useful for many laboratories worldwide using the VERSANT LiPA to genotype samples with undetermined results. We also decided to develop in-house sequencing assay for NS5B region because it is well recognized that NS5B is the most discriminant region for HCV genotyping.

## Methods

### Samples

In this retrospective study, we evaluated the determination of hepatitis C genotype of samples from HCV infected patients. In order to make the comparison of the sequencing of the 5’UTR, Core and NS5B regions we select 100 genotype pre-characterized samples from HCV infected patients.

Samples were selected to obtain at least 5 samples per genotype or G1 subtype except for G5 and G7, responsible for less than 2% of HCV infection in Belgium [3]. We also added undetermined VERSANT LiPA samples to challenge the sequencing assays.

All samples were received at the laboratory of Cliniques universitaires Saint-Luc for routine HCV genotyping and initially genotyped using our standard technique, the VERSANT HCV Genotype 2.0 Assay (VERSANT LiPA) (Siemens Healthcare Diagnostics, Erlangen, Germany). According to the LiPA protocol, the efficacy of the RT-PCR is not optimal for viral load below 2000 IU/mL, consequently, all analysed samples had a viral load above this limit.

No patient identifiers were included in the dataset used for this analysis. The study was approved by the local ethical committee.

### VERSANT HCV genotype 2.0 assay (LiPA)

After RNA extraction from plasma and RT-PCR of the 5’UTR and core regions of the HCV genome according to the manufacturer’s instructions, amplicons were obtained for 5’UTR and core regions. DNA products were hybridised with specific probes immobilised on a nitrocellulose membrane according to the manufacturer’s instructions. The hybridisation profile of the sample enables the genotype to be identified. The 5’UTR region is used for identification of the genotype and the core regions to specify the subtype of genotype 1. This assay can identify 6 genotypes and 18 subtypes (1a, 1b, 2a or c, 2b, 3a, 3b, 3c, 4a/c/d, 4b, 4e, 4f, 4 h, 5a, 6a or b). If the hybridisation profile was insufficient to identify a specific genotype, the genotype was reported as undetermined. When the hybridisation profile of the core region was insufficient, genotype 1 could not be discriminated from genotype 6.

### HCV RNA extraction and amplification of the HCV NS5B region

HCV RNA was extracted from a 200 μL of EDTA plasma sample with the Roche MagNA pure compact Nucleic Acid isolation kit I using the MagNA pure compact instrument (Roche Diagnostics, Basel, Switzerland), according to the manufacturer’s instructions. The extracted nucleic acid was eluted using 50 μL of elution buffer, HCV RNA was denaturised and reverse-transcribed. NS5B amplification was achieved using a KOD Hot Start DNA polymerase (Merck, Darmstadt, Germany) and specific NS5B amplification-FOR and REV primers describe by Margall et al. [15] (Table 1). The PCR was conducted using the following protocol: 2 min at 95 °C, 40 cycles of 20 s at 95 °C, 10 s at 60 °C and 5 s at 70 °C, and finally 5 min at 70 °C.

**Table 1:** Primers used for amplification and sequencing [15]

NS5B- amplification-FOR	5′-TATGATACYCGCTGYTTYGACTC-3’
NS5B-amplification-REV	5′-GTACCTRGTCATAGCCTCCGTGAA-3’
NS5B- sequencing-FOR	5′-CTCAACSGTCACTGAGAGWGACAT-3’
NS5B-sequencing-REV	5′-CACGAGCATSGTGCAGTCCYGGAGC-3’
5’UTR-sequencing-FOR	5′-GCAACAGGGAAYYTDCCUGGTTGCTC-3’
5’UTR-sequencing-REV	5’CTATCAGGCAGTACCACAAGG-3’
CORE-sequencing-FOR	5′-GTGCCCCGGGAGGTCTCGTAG-3’
CORE-sequencing-REV	5′-CCAAGGGTACCCGGGCTG-3’

All the PCR products were confirmed using electrophoresis on agarose gel. The expected amplicon size was 367 bp. All the PCR products were purified using the QIAquick PCR purification Kit according to the manufacturer’s instructions (Qiagen GmbH, Hilden, Germany). To optimise the sequencing, all the PCR products were diluted to a 10 ng/μL concentration with elution buffer (EB). The concentration of DNA was measured using the spectrophotometer GeneQuantII (Biochrom, Holliston, USA).

### Sequencing of 5’UTR, core and NS5B regions

The sequencing was based on the Sanger technique and the BigDye X Terminator (Life Technologies, Carlsbad, California, USA) was used. The different primers used are listed in Table 1. The primers used for sequencing of 5’UTR and core regions were designed in our laboratory. The primers used for sequencing of NS5B regions were describe by Margall et al. [15]. The sequencing protocol was as follows: 2 min at 96 °C, 40 cycles of 20 s at 96 °C, 10 s at 50 °C and 5 s at 60 °C, and finally the sequences were maintained at 40 °C.

For NS5B sequencing, PCR products from EDTA plasma diluted in EB buffer were used. For core and 5′-UTR, the products obtained from the VERSANT HCV Genotype 2.0 Assay were used. One microlitre of amplicon was diluted with 9 μL of reaction mix (4.5 μL DEPX H2O, 1.5 μL sequencing buffer 5x, 1 μL RRmix and 2 μL of sequencing primer). The DNA sequence was determined using the ABI3500 Genetic Analyzer (Thermo Fisher Scientific-Applied Biosystems, Foster City, California, USA).

The sequences obtained were read and aligned with the Geneious Prime® 2019.0.1 (Biomatters Ltd., Auckland, New Zealand). A consensus sequence was generated for each sample and compared with the HCV genomic Basic Local Alignment Search Tool (BLAST) bank for determination of the HCV genotype and subtype. A threshold for similarity of minimum 85% was used to consider the genotype or subtype.

### Phylogenetic analysis

Nucleotide sequences were aligned with reference sequences available in GenBank using Geneious Prime® 2019.0.1. A phylogenetic tree was reconstructed using the maximum likelihood (ML) approach implemented in PhyML 3.0 [16] with automatic selection of the best fit evolutionary model of DNA substitution (GTR + G + I) using the Akaike information criterion (AIC). Branch support was obtained by approximate likelihood-ratio test (aLRT, SH-like) [17], a likelihood based alternative to the computationally intensive bootstrapping.

A second phylogenetic tree was constructed using Neighbor-joining method with genetic distances computed by Tamura Nei mode. Using Geneious Prime® 2019.0.1.

### Statistical analysis

Statistical analysis was performed using MedCalc for Windows, version 18.6 (MedCalc Software, Ostend, Belgium). The kappa statistic was used to estimate the agreement between the different genotyping assays.

### Ethics approval

The study was approved by the local ethical committee “Comité d’Ethique Hospitalo-Facultaire Saint-Luc – UCL” under number 2018/30AVR/197.

## Results

### Characteristics of analysed samples

One hundred samples included in the study were analysed by the VERSANT LiPA assay and gave the following results: 25 G1 (8 G1a, 17 G1b), 6 G2, 11 G3, 13 G4, and 10 G6. It was not possible to discriminate between G1 and G6 by the VERSANT LiPA assay for 8 samples and 27 had an undetermined genotype with this assay.

A total of 100 patients were included in the study among which 41 were sequenced for the 3 regions, 5’UTR, core and NS5B, 23 patients were sequenced for 5’UTR and Core regions and 36 were sequenced for NS5B region only. This repartition was made in function of the available matrix.

### Genotype and subtype analysis

#### VERSANT LiPA assay compared to 5’UTR region sequencing

Out of the 64 VERSANT LiPA amplicons included, 49 sequences could be analysed for the 5’UTR region. Out of these 49 sequences, 40 (81.6%) had the same genotypes identified with the VERSANT LiPA assay (Table 2): 15 samples were identified as G1, 5 samples as G2, 7 as G3, 10 as G4 and 3 as G6. Out of the 15 samples identified as G1 by the two assays, the G1 subtype identified was identical for 9 samples (2 G1a and 7 G1b). For the determination of the type and G1 subtype, the kappa coefficient was 0.94 and 0.82 respectively, which means that the strength of agreement between VERSANT LiPA assay and 5’UTR region sequencing was almost in perfect agreement. Of 7 samples for which discrimination between G1b and G6 was not possible with the VERSANT LiPA assay, 1 was identified as G6 by 5’UTR sequencing, 5 as G1b and 1 sample remained undetermined. Of the 9 samples identified as G6 by the VERSANT LiPA assay, 3 were also identified as G6 by 5’UTR sequencing, 1 as G1b and 4 were undetermined (Table 3).

**Table 2:** Concordance for HCV genotype and subtype determination with the different genotyping assays – VERSANT LiPA assay as reference

Genotype	Concordance percentage: VERSANT LiPA assay vs		
	5’UTR sequencing (n)	Core sequencing (n)	NS5B sequencing (n)
G1	100% (15/15)	94% (15/16)	92% (12/13)
G1a	40% (2/5)	75% (3/4)	100% (3/3)
G1b	70% (7/10)	100% (12/12)	90% (9/10)
G2	100% (5/5)	100% (5/5)	100% (6/6)
G3	100% (7/7)	86% (6/7)	100% (7/7)
G4	91% (10/11)	100% (10/10)	100% (8/8)
G6	60% (3/5)	100% (8/8)	100% (3/3)
G1 or 6	100% (6/6)	100% (7/7)	100% (5/5)

*G* Genotype

**Table 3:** Genotype with the four assays for samples for which discrimination between G1 and G6 was not possible with the VERSANT LiPA assay

Sample number	Genotype			
	VERSANT LiPA assay	5’UTR sequencing	CORE sequencing	NS5B sequencing
HCV066	1b or 6	NI	1b	1
HCV067	1b or 6	1b	1b	1b
HCV068	1b or 6	1b	1b	1b
HCV069	1b or 6	1b	1b	NR
HCV070	1b or 6	1b	6	6
HCV071	1b or 6	1b	1b	NI
HCV072	1b or 6	6	6	NR
HCV073	1a or 6	NR	NR	1

*NI* sequence not interpretable, *NR* not realised (short sample)

All discordant samples are detailed in the Table 4.

**Table 4:** Genotype with the four assays for samples discordant between the assays

Sample number	Genotype			
	VERSANT LiPA assay	5’UTR sequencing	CORE sequencing	NS5B sequencing
HCV001	1a	1a/b	1a	1a
HCV003	1a	1a/b	1a	NR
HCV004	1a	1b	6	NR
HCV010	1b	1a/b	1b	1b
HCV011	1b	1a/b	1b	1b
HCV013	1b	NI	1b	6
HCV020	1b	1	1b	NR
HCV037	3a	3	3/2	NR
HCV046	4	4/3	4	NR
HCV060	6	1b	6	NR
HCV062	6a/6b	1b/6	6	NR
HCV066	1b*	NI	1b	1
HCV067	1b*	1b	1b	1b
HCV068	1b*	1b	1b	1b
HCV069	1b*	1b	1b	NR
HCV070	1b*	1b	6	6
HCV071	1b*	1b	1b	NI
HCV072	1b*	6	6	PA
HCV073	1a ou 6	NR	NR	1
HCV075	ID	1/3/6	6	1b
HCV076	ID	1b	1b	6
HCV001	1a	1a/b	1a	1a
HCV003	1a	1a/b	1a	NR
HCV004	1a	1b	6	NR
HCV010	1b	1a/b	1b	1b
HCV011	1b	1a/b	1b	1b
HCV013	1b	NI	1b	6

*NI* sequence not interpretable, *NR* not realised (short sample), *UND* Genotype undetermined by the VERSANT LiPA assay

#### VERSANT LiPA assay compared to core region sequencing

Out of the 64 VERSANT LiPA amplicons included, 56 sequences could be analysed for the Core region. Out of these 56 sequences, 44 (78.6%) had the same genotype identified with the VERSANT LiPA assay (Table 2): 15 samples were identified as G1, 5 samples as G2, 6 as G3, 10 as G4 and 8 as G6. Out of the 15 G1 samples, both assays revealed the same for all samples (3 G1a and 12 G1b). For the determination of the type and G1 subtype, the kappa coefficient was 0.92 and 1 respectively, which means that the strength of agreement between VERSANT LiPA assay and core region sequencing was almost in perfect agreement.

Of 7 samples for which discrimination between G1b and G6 was not possible with the VERSANT LiPA assay, 2 were identified as G6 by Core sequencing and 5 as genotype 1b. Of the 9 samples identified as G6 by the VERSANT LiPA assay, 8 were also identified as G6 with core sequencing and 1 was undetermined (Table 3).

All discordant samples are detailed in the Table 4.

#### VERSANT LiPA genotyping compared to NS5B region sequencing

Out of the 77 EDTA samples analysed for the NS5B region only 69 (89,6%) were correctly amplified and used in this comparison. Thirty-six (52,2%) samples had the same genotype identified with the VERSANT LiPA assay: 12 samples were identified as G1, 6 as G2, 7 as G3, 8 as G4 and 3 as G6. Out of the 12 samples identified as G1 by the two assays, the G1 subtype identified is the same for all the samples (3 G1a and 9 G1b). For the determination of the type and G1 subtype, the kappa coefficient was 0.94 and 1 respectively, which means that the strength of agreement between VERSANT LiPA assay and NS5B region sequencing was almost in perfect agreement.

Of 5 samples for which discrimination between G1 and G6 was not possible by the VERSANT LiPA assay, 1 was identified as G6 by NS5B sequencing, 2 as G1b and 2 as G1 without subtype information. Of the 3 samples identified as G6 by the VERSANT LiPA assay, all were confirmed as G6 with NS5B sequencing.

All discordant samples are detailed in the Table 4.

#### NS5B region sequencing compared with the other assays

Knowing that the sequencing of the NS5B region is the genotyping method currently considered to be the gold standard, we have presented the results also according to the genotype given by this sequencing. Table 5 allows us to assess the concordance of the different assays. In our sample cohort, we can see that the agreement between the different assays is 100% for G2, G3 and G4. The percentage of agreement decreases for G1 and G6. For G1, the worst agreement is reported for the VERSANT LiPA assay with a percentage of 75% followed by the sequencing of the 5′ UTR region with a percentage of 85.7%. The sequencing of the core region shows the best agreement with a percentage of 89%. We can notice that this mismatch is due to the misidentification of the G1b for all the tests (3 samples out of 6 correctly identified with the sequencing of the 5′ UTR region, 9 samples out of 11 with the VERSANT LiPA and 6 samples out of 7 with the sequencing of the core region).

**Table 5:** Concordance for HCV genotype and subtype determination with the different genotyping assays – NS5B sequencing as reference

NS5B	Concordance percentage: NS5B sequencing vs		
	5’UTR sequencing (n)	Core sequencing (n)	VERSANT LiPA assay (n)
G1	85,7% (6/7)	89% (8/9)	75% (12/16)
G1a	0% (0/1)	100% (1/1)	100% (3/3)
G1b	50% (3/6)	86% (6/7)	82% (9/11)
G2	100% (6/6)	100% (5/5)	100% (6/6)
G3	100% (3/3)	100% (3/3)	100% (7/7)
G4	100% (6/6)	100% (5/5)	100% (8/8)
G6	0% (0/2)	60% (3/5)	60% (3/5)
G2k/1b	0% (0/1)	0% (0/1)	–

*G* Genotype

### Phylogenetic analysis

**Fig. 1:**
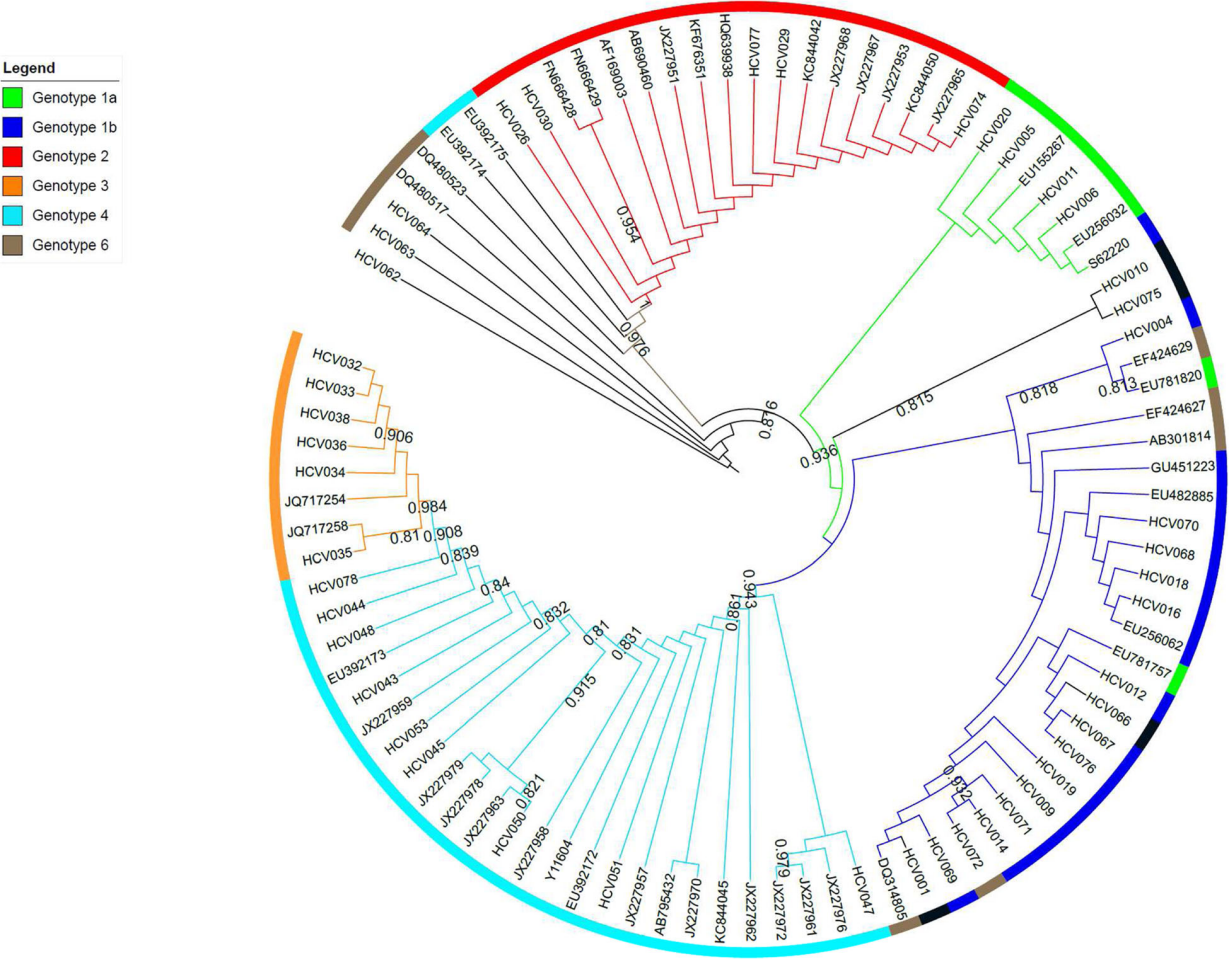
Maximum likelihood phylogenetic tree (ML) of the 5’UTR gene sequences from 47 reference sequences and 45 sample sequences. The tree branches, including the outer circle, are colored according to the HCV types and subtypes 1a and 1b. The tree scale refers to the number of nucleotide substitutions per site. ML tree showed that genotyping based on 5’UTR sequencing was the least discriminating showing erroneous clustering mainly with respect to G1a (in green in the figure), G1b (in blue) and G6 (in brown). We can also observe that G4 (in light blue) is divided into 2 groups

**Fig. 2:**
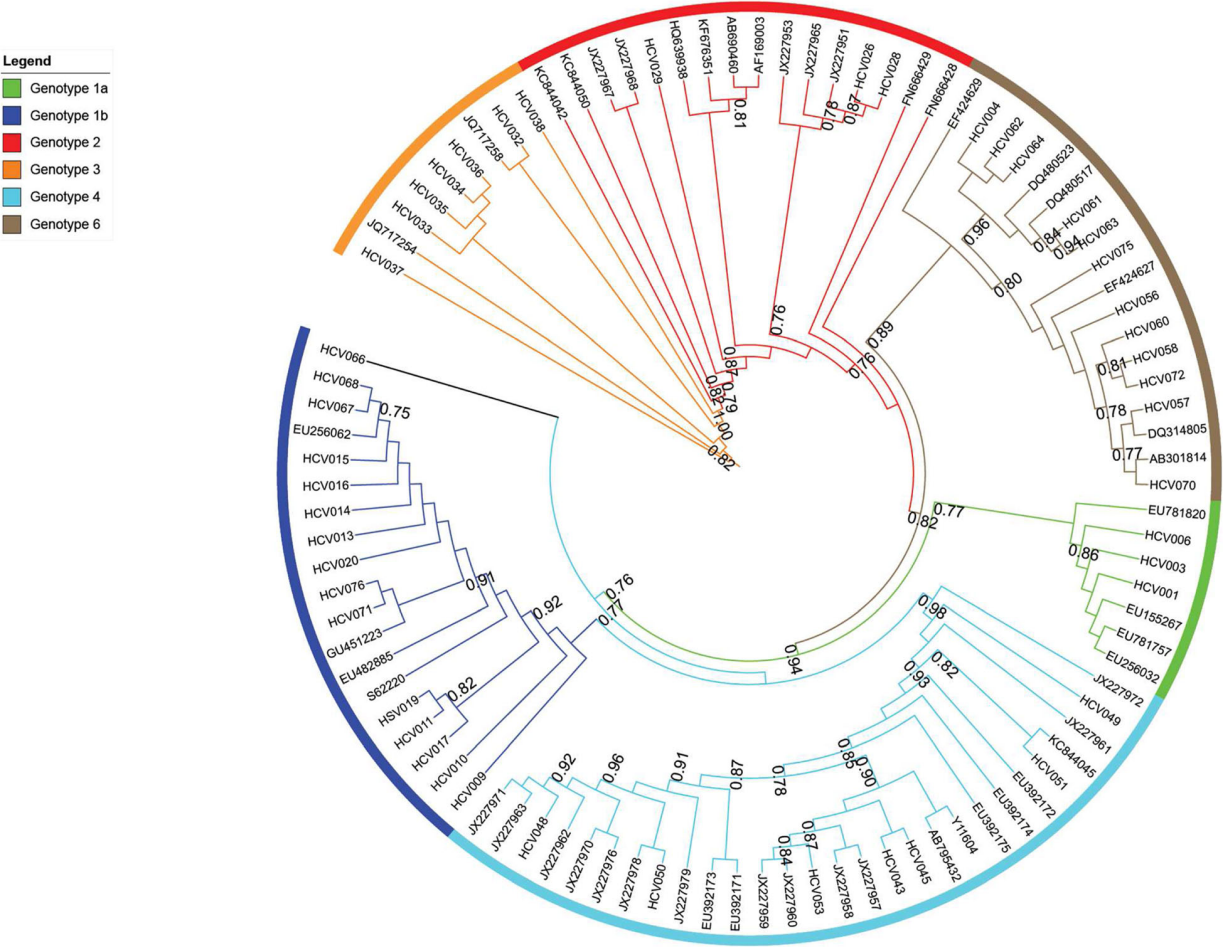
Maximum Likelihood (ML) phylogenetic tree of the Core gene sequences from 47 reference sequences and 50 sample sequences. The tree branches, including the outer circle, are colored according to the HCV types and subtypes 1a and 1b. The tree scale refers to the number of nucleotide substitutions per site. The ML tree of the Core region showed a very good grouping of the different genotypes, for reference and sample sequences

**Fig. 3:**
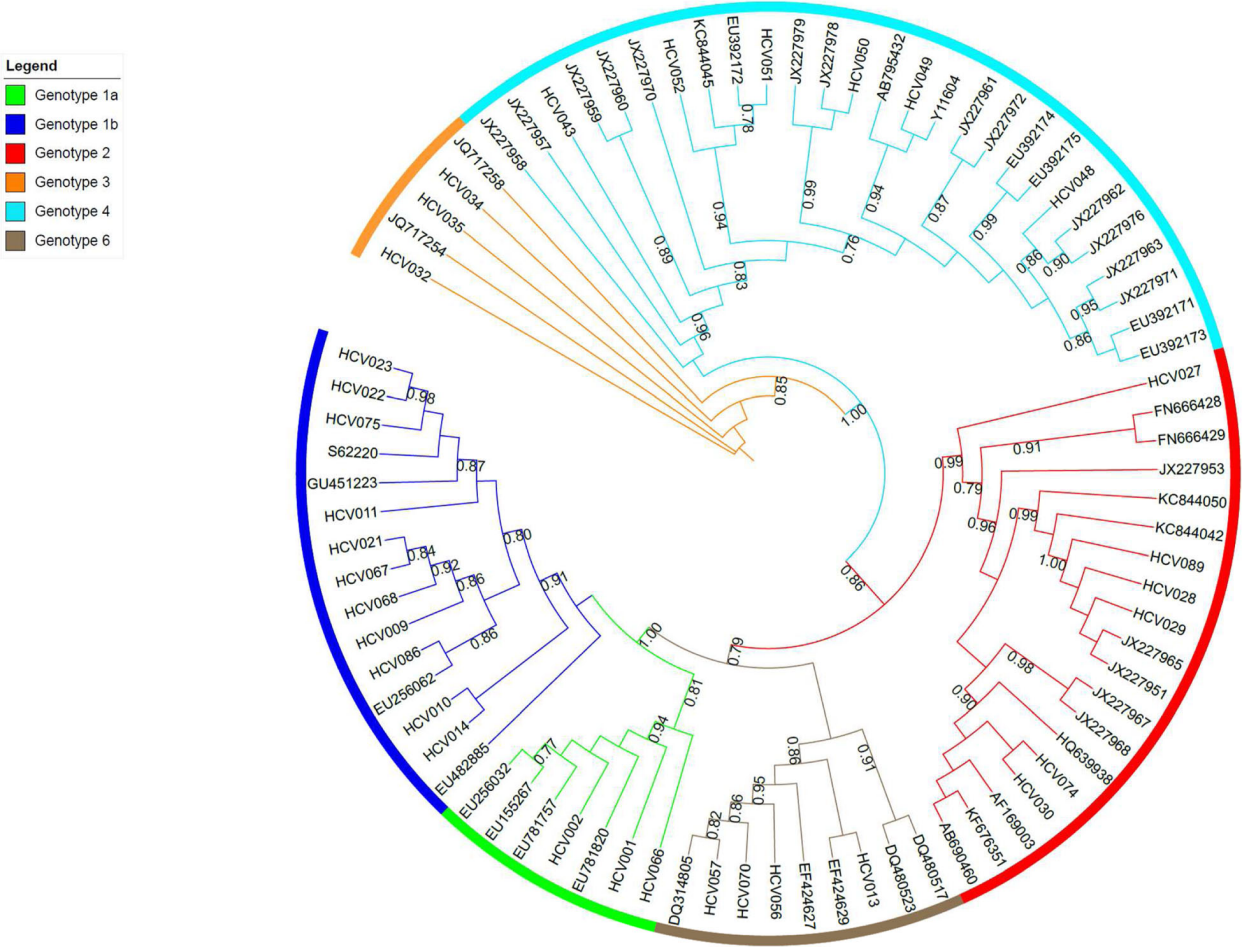
Maximum Likelihood (ML) phylogenetic tree of the NS5B gene sequences from 47 reference sequences and 35 sample sequences. The tree branches, including the outer circle, are colored according to the HCV types and subtypes 1a and 1b. The tree scale refers to the number of nucleotide substitutions per site. The ML tree of the NS5B region showed the best grouping of the different genotypes, for reference and sample sequences. This discrimination even extended to the subtype for instance for G4 in light blue in the Figure

Phylogenetic trees were build using 47 reference sequences and 45 sample sequences for the 5’UTR region, 50 sample sequences for the Core region and 35 sample sequences for the NS5B region. Maximum likelihood tree (Figs. 1, 2 and 3) showed that genotyping based on the 5’UTR sequencing was the less discriminant compared with genotyping based on Core or NS5B sequencing. Indeed, when we look at the 5′ UTR tree (Fig. 1), we can see that the genotypes mix mainly with regard to G1a, G1b and G6. We can also notice that G4 was split into two groups. On the contrary, the trees of the Core region (Fig. 2) and NS5B region (Fig. 3) shown a very good grouping of the different genotypes, both for the reference sequences and the sample sequences. This discrimination even extends to the subtype (clearly visible for the G4) for the NS5B tree (Fig. 3).

Trees obtained from Neighbor-joining method showed similar results (date not shown).

## Discussion

Following the arrival of the newer approved anti-HCV pangenotypic DAAs, one might think that genotype determination is of little use, however, according to the last EASL recommendations [5] for the Treatment of Hepatitis C published in 2018, determining the HCV genotype, including subtypes 1a and 1b, remains useful when choosing a treatment and its duration. Assays that are recommended for genotyping are those that can discriminate genotyping/subtyping with amplification of the 5’UTR region plus one other region, usually the NS5B or core region. Commercial assays perform these goals with a globally good performance [18]. The two most frequently used commercial assays are the VERSANT LiPA and GII Abbott assays. Regardless of the assay used, discriminating between genotype 6 and genotype/subtype 1a and 1b remains a challenge. In approximately 5% of samples, this distinction cannot be made [10]. For the GII Abbott assay, more than 90% of genotypes and subtypes would be correctly identified [10, 11, 18, 19] and Mallory et al. [11] recommended to confirm samples with coinfected genotypes or an unkown G1 subtype with another method, representing 5.5% of samples included in their cohort. The VERSANT LiPA failed to distinguish the subtype of genotype 1b in 17 samples (6%) of 276 samples over a 2-year period in our hospital. During the same period, 24 (3.5%) of 678 genotypes performed could not be determined. These percentages are concordant with the literature [14, 19–21].

In this study, we investigated the accuracy of the VERSANT LiPA assay for genotyping HCV with sequencing of three different regions of the HCV: the 5’UTR, the core region and the NS5B region. Sequencing of the NS5B region was performed on EDTA plasma samples while sequencing of the 5’UTR and core regions was performed from VERSANT LiPA amplicons. One hundred samples were included and all were pre-genotyped by the VERSANT LiPA assay. Sixty-four VERSANT LiPA amplicons were sequenced for 5’UTR and core regions. Seventy-seven EDTA plasma samples were sequenced for the NS5B region. We showed that the amplification rate was the lowest for NS5B, only 69 samples out of 77 (89,6%) were correctly amplified, this low rate of amplification is probably explained by the fact that most of the samples included in this study were complicated samples. In addition, matrices used have undergone higher freeze-thaw cycles than a routine cohort. It is well known that the NS5B region is less preserved than the Core or 5’UTR region but in contrast, it allows the most correct HCV genotype determination [22].

The HCV genotypic distribution of the samples included in the study was not representative of the HCV genotypic prevalence in Belgium; indeed the samples included were preselected to have a mix of the different genotypes found in our country.

When taking the VERSANT LiPA assay as a reference, we showed that the global accuracy for genotyping was high if undetermined genotypes were not taken into account; globally, accuracy was greater than 95%. If we take the sequencing of the NS5B region as reference, this global accuracy fell. However, sequencing enabled discrimination of all the samples (27/100) that were not identified by the VERSANT LiPA assay. Genotype/subtype 1a, 1b and 6 were accurately identified in more than 95% of samples with sequenced Core and NS5B regions, but this percentage was lower for 5’UTR region sequencing with accurate identification in only 69% of samples. These values are concordant with those reported in the literature [10, 11, 14, 18–21]. Indeed, it is well established that sequencing of the 5’UTR region of HCV is not sufficient to distinguish between genotype/subtypes 1a, 1b and 6 [9].

Few studies have shown that the genotyping challenge is higher for the subtyping of G1a and 1b [23–25]. Knowing that G1a and G1b are associated with different rates of resistance-associated variants and different responses to DAAs, correct identification of G1 subtype remains important and may have implications for responses rate to DAAs [25, 26].

Based on the literature and our own data, we would be very cautious in interpreting HCV genotyping result if the Abbott test is used in the first line and we would not hesitate to do the NS5B sequencing assay as a confirmation test. When using the VERSANT LiPA assay, G1 subtyping without core region information should be confirmed by a sequencing method. All undetermined results must also be sequenced. HCV genotyping results that would be confirmed represent approximately 5% in our cohort. Taking in account the cost of these confirmation tests, we report here an assay that uses VERSANT LiPA amplicons already available allowing a significant saving of costs, workload, set up of a RT-PCR steps and would not require extra plasma samples.

We would therefore recommend the sequencing of the core region when the VERSANT LiPA amplicons are available and the sequencing of the NS5B region starting from plasma sample. As shown in the analysis of phylogenetic trees, the sequencing of the NS5B region allows for better discrimination of HCV genotyping and subtyping, the sequencing of the core region gives results close to those of NS5B sequencing, the 5′ UTR region is the least recommended of the 3 regions due to low discrimination. Homemade or commercial Sanger sequencing or Next Generation sequencing assays should be performed at least in specialized laboratories such as National Reference Centers.

## Conclusions

The high genetic variability of HCV makes it a challenge to correctly determine genotype and subtypes using commercial assays. For undetermined samples, supplementary testing is required. Even if no G5 and G7 were included in our study, we describe new and original methods of sequencing 5’UTR and Core regions to confirm HCV genotypes not discriminated by a commercial assay, in particular by using amplicons already obtained by the VERSANT HCV Genotype 2.0 Line Probe Assay. This method thus saves costs and workload of RT-PCR steps if a confirmation assay is needed and might be of usefulness for overcoming the problem of undetermined results in many laboratories worldwide performing the VERSANT LiPA assay.

## Data Availability

The complete datasets used and analysed during the current study are available from the corresponding author on reasonable request.

## Abbreviations

5’UTR: 5’Untranslated region
Abbott GTII: Abbott RealTime HCV Genotype II
DAA: direct acting agents
G: Genotype
HCV: Hepatitis C Virus
VERSANT LiPA: VERSANT HCV Genotype 2.0 Line Probe Assay
